# R Statistics: survey and review of packages for the estimation of Rasch models

**DOI:** 10.5116/ijme.629d.d88f

**Published:** 2022-06-24

**Authors:** John M. Linacre

**Affiliations:** 1 Research Director, Winsteps.com, USA

**Keywords:** R Statistics, R packages, estimation of Rasch models, large Dichotomous Data Frame

## Abstract

Abstract - R Statistics is a comprehensive and widely-used suite of packages for statistical operations. From 27 R packages indexed with the word “Rasch”, 11 packages capable of Rasch estimation and analysis are identified and critiqued. A commercial Rasch application is included for comparison. Three R data frames are used. A larger and a smaller 0/1 data frame are analyzed with the Dichotomous Rasch Model. A polytomous 0/1/2 data frame is analyzed with the Partial Credit Model. The R packages can all use the same data frame. They are easy to use and mostly fast, though their documentation is generally skimpy. Every package has obvious shortcomings, but the unique features of each package could make them all useful. For general Rasch estimation and fit analysis of dichotomous data, three packages stand out: eRm, TAM and autoRasch. Two packages stand out for polytomous data: TAM and autoRasch.

## Introduction

R Statistics is a comprehensive collection of software packages for statistical analysis. These packages are developed and contributed by volunteers, and then made available to anyone. Some packages are intended to be long-term and authoritative. Other packages are implemented for one project, contributed to R Statistics by their authors, and then the authors move on to other projects. Advantages of R packages are that they are free and immediately available, and all packages use the same data file and instruction formats. The disadvantages are that their documentation may be incomplete. They usually have no support from their authors, and bugs in the software may not be remedied. As of this writing, there are over 2 million R users^1^and more than 18,000 R packages.^2^ Many R packages perform similar functions. It can be difficult to identify which packages best meet your needs.

Here I evaluate R packages for estimating person ability (thetas, rows in the data frame) and item difficulty (betas, columns in the data frame) by means of Rasch analysis. The words “ability” and “difficulty” are for convenience. Different words apply in different contexts, but the underlying algebra remains the same. A search of R Statistics packages with the word “Rasch” in their titles or descriptions (metadata) was conducted using METACRAN.^3^ This produced the names of 27 R packages. 13 of these packages do not perform Rasch estimation: DIFboost, DIFlasso, pwrRasch, RaschSampler, Rwinsteps, scaleAlign, tcl, birtr, iarm, mixRaschTools, OPDOE, dscore, whomds. Packages psychomix and psychotree are conveniently accessed through package psychotools. Package RM.weights is not recommended because it crashed on the 0/1 data frame described below.

Several of the 11 remaining packages support more than one method of estimating Rasch measures. In general, the estimation method more prominent in the package documentation is chosen. An exception is package sirt which implements many estimation methods. Three of these are included. Further, a commercial Rasch software package, Winsteps,^4^is included for comparison purposes with two estimation methods.

For practical use, we are looking for packages than can estimate Rasch person and item measures (parameter values) for dichotomous, binary, 0/1 data and also for polytomous, rating scale, 0/1/2/… data using the Partial Credit Model. The packages also need to support quality-control fit analysis for individual persons and items. To assist with this, three R data frames were constructed, representing the typical use of the Rasch model.

## Estimation with a large Dichotomous Data Frame

**Figure 1:**
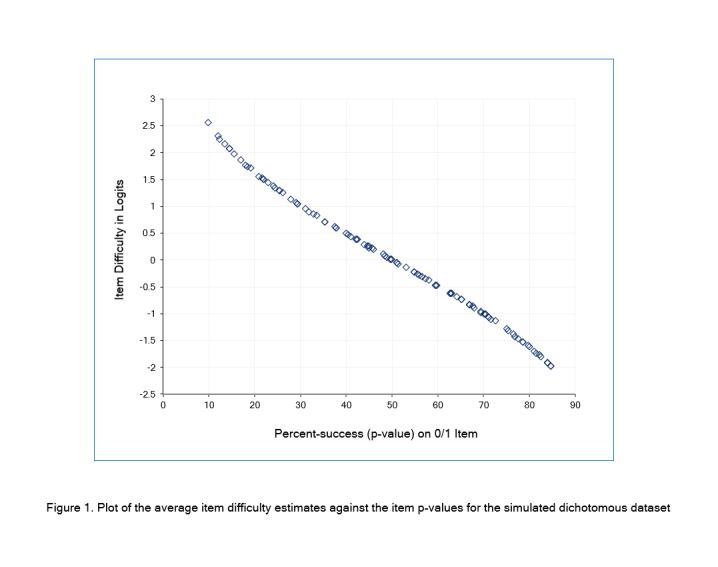
Plot of the average item difficulty estimates against the item p-values for the simulated dichotomous dataset

A rectangular dataset^5^ of 105 items (columns) and 200 persons (rows) containing dichotomous 0/1 data was simulated to fit the Rasch model. 5 of these items deliberately have some misfit. Also, no item or person has an extreme score of all 0s or all 1s. There is no missing data. There are no person identifiers in the data frame. This dataset was loaded into an R data frame and analyzed by the Dichotomous Rasch Model (RM). In addition, the packages were checked for their robustness against extreme scores and missing data. This investigation was conducted using R version 4.1.3. Table 1 lists the 15 estimation options considered here. Item difficulties (betas) were estimated by each package and adjusted so that the mean value of each set of item difficulties was zero. The 15 estimates of each item difficulty were then averaged. For all the packages, the correlation between their set of item difficulties and the set of average difficulties was 1.000. The relationship between the average estimates in logits and the percent-success (p-value) on the items is shown in Figure 1. The curve has the expected curvilinear monotonic shape. The x-axis has the possible range of 0-100, and the y-axis is infinite in both directions.

**Table 1:** R Packages for Rasch Estimation from Dichotomous Data


Package	Version	Beta	Extreme		Missing data	Dichotomous analysis	Theta output
			item	person			
autoRasch	0.1.5	JMLE	‡	✔	✔	res <- pcm(data)	res$theta
eRm	1.0-2	CMLE	†	†	✔	res <- RM(data)	summary(person.parameter(res))
ltm	1.2-0	MMLE	✔	✔	✔	res <- rasch(data, constraint = cbind(ncol(data) + 1, 1))	✘factor.scores(res)
mixRasch	1.1	JMLE	†	✔	✔	res <- mixRasch(data,1,50, conv.crit=.0001, n.c=1)	res$person.par$theta
pairwise	0.5.0-2	PMLE	✔	✔	✔	✘res <- pair(daten = data, m = 2)	✘summary(pers(res))
pcIRT	0.2.4	CMLE	‡	✔	✘	res <- DRM(data)	✘
plRasch	1.0	LLA	✔	✔	✘	res <- RaschPLE(data, rep(1, ncol(data)), 1)	✘
psychotools	0.7-1	CMLE	†	†	✔	res <- raschmodel(data)	✘personpar(res)
sirt	3.11-21	MMLE	‡	✔	✔	res <- rasch.mml2(data)	wle.rasch(dat=data, b=res$item$b)
sirt	3.11-21	PMLE	†	✔	✔	✘res <- rasch.pairwise(data)	(as above)
sirt	3.11-21	VA	‡	✔	✘	res <- rasch.va(data)	(as above)
TAM	3.7-16	MMLE	‡	✔	✔	res <- tam.mml(data)	tam.wle(res)$theta
tmt	0.3.0-20	CMLE	†	†	✔	res <- tmt_rm(data)	✘
Winsteps	5.2.2	CMLE	✔	✔	✔	Excel/RSSST menu	(same)
Winsteps	5.2.2	JMLE	✔	✔	✔	Excel/RSSST menu	(same)


✔ = performance is satisfactory ✘ = performance is not available, failed or is not satisfactory † = Analysis proceeds, but extreme items or persons are omitted or ignored ‡ = Analysis fails. Extreme items must be removed from data frame. CMLE = Conditional Maximum Likelihood Estimation; JMLE = Joint MLE; MMLE = Marginal MLE; PMLE = Pairwise MLE; LLA = Log-Linear Association; VA = Variational Approximation

**Figure 2:**
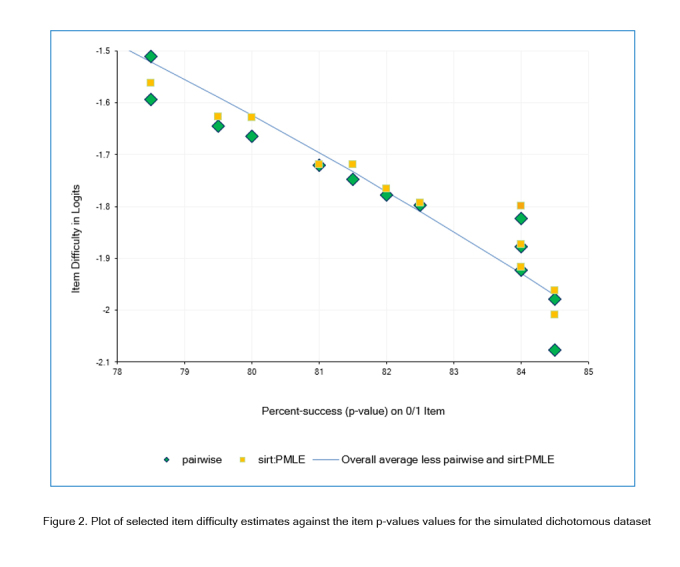
Plot of selected item difficulty estimates against the item p-values values for the simulated dichotomous dataset

Most item estimates were less than .01 logits away from the corresponding overall average estimates. However, some of the estimates made by sirt:PMLE and pairwise were more than 0.1 logits away from the overall average, as shown in Figure 2. Also, for each of these two estimators, some items with the same p-value (x-axis) have different logit estimates (y-axis), as shown by the same icon appearing more than once in a vertical column in Figure 2. This is a feature of PMLE (Pairwise maximum Likelihood Estimation) but is contrary to the general Rasch principle that “the same total score by the same persons (no missing data) produces the same item difficulty, regardless of the items' response patterns.”

The next consideration is the estimation of person measures (thetas). Packages pcIRT, plRasch and tmt did not produce these. Packages ltm, pairwise and psychotools did not output thetas in a format directly suitable for fit analysis (or, at least, not that I could determine from their documentation). Each person measure is estimated using the package’s set of logit item difficulties and the person’s raw score. Since the item difficulties are almost the same for the 9 remaining estimation options, the differences between the estimated person measures are expected to be small. The standard deviations of the 9 sets of thetas were between 0.966 and 0.980 logits, and so essentially the same despite packages implementing different theta estimators.

In practical situations, datasets may have items or persons with extreme scores and may contain missing data (R Statistics code “NA”). Omitting or ignoring items with extreme scores from the output may be annoying but is unlikely to be misleading. Omitting or ignoring persons with extreme scores may lead to misleading summary statistics and so incorrect inferences later. The inability to analyze data frames with missing data may require a change of Rasch package at an inconvenient time. Package capabilities for these are shown in Table 1, and incapable packages are dropped from further consideration.

Differences between Rasch options are more pronounced with smaller datasets. Accordingly, an R data frame was constructed from the “Knox Cube Test” (KCT) Ref.6 0/1 data, omitting items and persons with extreme scores and with no missing data. This has 14 items and 34 persons. The item estimates for the 8 packages all correlate 1.00 with the average of all 8 estimates. Table 2 shows that the item estimates for the JMLE Packages are somewhat more dispersed than the CMLE and MMLE estimates. This indicates that mixing estimates produced by different R packages is to be avoided. When they are accumulated together, they must be linearly transformed so that they are all on the same measurement scale, similarly to the same way that inches and centimeters are rescaled when they are combined.

**Table 2:** R Packages for Rasch Estimation from Dichotomous Data (Knox Cube Test) and PCM data (Liking for Science)

Package	Item Estimation Method	Item Estimate S.D. for KCT	Item Estimate S.D. for PCM	Diagnostic Fit Statistics		Recommended R Packages	
				Items	Persons	Dichotomous	PCM
eRm	CMLE	3.04	*1.71*	✔	✔	✔	
sirt	MMLE	3.09	1.20	✘	✘		
TAM	MMLE	3.09	1.20	✔	✔	✔	✔
Winsteps	CMLE	3.13	1.19	✔	✔		
mixRasch	JMLE	3.15	1.25	✔	✘		
autoRasch	JMLE	3.26	1.23	✔	✔	✔	✔
Winsteps	JMLE	3.49	1.25	✔	✔		
sirt	VA	3.60	✘	✘	✘		

✔ = performance is satisfactory ✘ = performance is not available, failed or is not satisfactory

An essential step in the Rasch analysis of empirical data is investigating the fit of the responses by each person and to each item. Three R packages provide satisfactory estimation and fit analysis for a dichotomous data set: autoRasch, eRm and TAM.

## Polytomous data: PCM

A rectangular dataset of 24 items and 74 persons containing 0/1/2 data was extracted from the “Liking for Science”^7^ datasets. Again extreme scores were omitted, and also all 3 categories were observed on every item. This dataset is analyzed by the Partial Credit Model (PCM). Table 2 shows 7 R packages capable of meaningful PCM analysis with a 3-category rating scale. The item estimates for 6 packages correlate 1.000 with the average of all the packages. Package eRm correlates to 0.993 and also has a noticeably larger item estimate S.D. than the other packages. Consequently, eRm is not recommended for polytomous data. Packages autoRasch and TAM are recommended for PCM analysis.

## Discussion

It is no surprise that eRm (more than 100,000 downloads from the RStudio CRAN mirror)^8^ and TAM (more than 250,000 downloads) are among the most capable Rasch R packages. They are widely used. AutoRasch (more than 1,000 downloads) is a new package released in 2022, designed to have special functions, but which is also very capable in standard situations. In fact, each Rasch R package has unique features. These may make a different package more suitable for your application. However, the generally skimpy documentation of the packages can make their use more challenging, and expect to have to work around any programming bugs. Advantages of these packages include that they can all analyze the same. rdata data frames. Their output can also be displayed with R plotting packages, such as WrightMap, though this may take some effort the first time. In general, using these R packages is easy and fast.

### Conflict of Interest

The author declares that they have no conflict of interest.
